# Sunitinib efficacy with minimal toxicity in patient-derived retinoblastoma organoids

**DOI:** 10.1186/s13046-023-02608-1

**Published:** 2023-02-01

**Authors:** Atthapol Srimongkol, Natanan Laosillapacharoen, Duangporn Saengwimol, Vijender Chaitankar, Duangnate Rojanaporn, Thanastha Thanomchard, Suparerk Borwornpinyo, Suradej Hongeng, Rossukon Kaewkhaw

**Affiliations:** 1 Research Center, Faculty of Medicine Ramathibodi Hospital, Mahidol University, 10400 Bangkok, Thailand; 2 Program in Translational Medicine, Faculty of Medicine Ramathibodi Hospital, Mahidol University, 10400 Bangkok, Thailand; 3 Biodata Mining and Discovery Section, National Institute of Arthritis and Musculoskeletal and Skin Diseases, National Institutes of Health, Bethesda, MD USA; 4 Department of Ophthalmology, Faculty of Medicine Ramathibodi Hospital, Mahidol University, 10400 Bangkok, Thailand; 5 Ramathibodi Comprehensive Cancer Center, Faculty of Medicine Ramathibodi Hospital, Mahidol University, 10400 Bangkok, Thailand; 6 Excellent Center for Drug Discovery, Faculty of Science, Mahidol University, 10400 Bangkok, Thailand; 7 Department of Biotechnology, Faculty of Science, Mahidol University, 10400 Bangkok, Thailand; 8 Department of Pediatrics, Faculty of Medicine Ramathibodi Hospital, Mahidol University, 10400 Bangkok, Thailand; 9 Chakri Naruebodindra Medical Institute, Faculty of Medicine Ramathibodi Hospital, Mahidol University, 10540 Samut Prakan, Thailand

**Keywords:** Sunitinib, Retinoblastoma, Drug screening, Organoid, Retinal toxicity

## Abstract

**Background:**

Recurrence of retinoblastoma (RB) following chemoreduction is common and is often managed with local (intra-arterial/intravitreal) chemotherapy. However, some tumors are resistant to even local administration of maximum feasible drug dosages, or effective tumor control and globe preservation may be achieved at the cost of vision loss due to drug-induced retinal toxicity. The aim of this study was to identify drugs with improved antitumor activity and more favorable retinal toxicity profiles *via* screening of potentially repurposable FDA-approved drugs in patient-derived tumor organoids.

**Methods:**

Genomic profiling of five RB organoids and the corresponding parental tissues was performed. RB organoids were screened with 133 FDA-approved drugs, and candidate drugs were selected based on cytotoxicity and potency. RNA sequencing was conducted to generate a drug signature from RB organoids, and the effects of drugs on cell cycle progression and proliferative tumor cone restriction were examined. Drug toxicity was assessed with human embryonic stem cell-derived normal retinal organoids. The efficacy/toxicity profiles of candidate drugs were compared with those of drugs in clinical use.

**Results:**

RB organoids maintained the genomic features of the parental tumors. Sunitinib was identified as highly cytotoxic against both classical *RB1*-deficient and novel *MYCN*-amplified RB organoids and inhibited proliferation while inducing differentiation in RB. Sunitinib was a more effective suppressor of proliferative tumor cones in RB organoids and had lower toxicity in normal retinal organoids than either melphalan or topotecan.

**Conclusion:**

The efficacy and retinal toxicity profiles of sunitinib suggest that it could potentially be repurposed for local chemotherapy of RB.

**Supplementary Information:**

The online version contains supplementary material available at 10.1186/s13046-023-02608-1.

## Background

Retinoblastoma (RB), a tumor of the developing retina, is the most frequent primary intraocular cancer in children and has an estimated worldwide incidence of 8000 cases annually [1]. The advent of chemotherapy, first systemically and then locally *via* intra-arterial or intravitreal administration, has led to improved treatment outcomes and great success in globe retention [2–7]. However, some tumors are resistant to even local administration of maximum feasible drug dosages, and surgical removal of the eye becomes necessary to prevent death associated with extraocular dissemination [2, 3, 6]. In other cases, tumor control is effective and the globe can be preserved, but at the cost of drug-induced retinal toxicity and loss of vision [8–12]. Thus, there is an urgent need to identify drugs with improved antitumor activity and more favorable retinal toxicity profiles for the treatment of RB.

Drug repurposing (also known as repositioning or reprofiling) is receiving increased interest as an alternative and more cost-effective strategy compared with *de novo* drug development. However, preclinical testing of drugs is still necessary to avoid unexpected lack of efficacy and/or unnecessary side effects during treatment for a new indication. Tumor organoids derived from patient tissues have been reported to faithfully model many aspects of the parent tumor and to serve as effective *in vitro* models for testing of anticancer agents [13–17]. In many studies, organoid cultures have been shown to recapitulate the genomic and transcriptomic profiles of donor tumors and be predictive of patient-drug responses [13, 16]. Additionally, differences in drug responses associated with intratumoral heterogeneity has been demonstrated in patient-derived organoids [14, 15, 17].

Tumor organoids representing two known subtypes of RB have been reported and shown to exhibit tumor-relevant genotypes and phenotypes [18, 19]. Classical RB is initiated by biallelic inactivation of the tumor suppressor gene *RB1* and identified in most RB cases, whereas novel subtype of RB is initiated by high focal *MYCN* amplification and accounts for < 2% of all cases [20, 21]. *RB1*-deficient and *MYCN-*amplified RBs appear to differ with respect to genetic and epigenetic characteristics that lead to differences in expression levels of cone markers and in stemness [20, 22–24]. Given these differences, it is possible that the RB subtypes may display differing responses to the same chemotherapy regimen, and whether a single drug could be developed to treat both RB subtypes is currently unknown.

In this study we aimed to discover whether one or more FDA-approved anticancer drugs could potentially be repurposed for the treatment of RB. We performed high-throughput screening of 133 FDA-approved drugs on fully characterized patient-derived RB organoids and conducted detailed mechanistic investigations of selected candidates. We compared the efficacy and toxicity profiles of candidate drugs in *RB1*-deficient and *MYCN-*amplified RB organoids and in retinal organoids derived from human embryonic stem cells (hESCs) with those of clinically used anticancer drugs.

## Methods

### Human tissues

RB was located *via* transillumination of the enucleated eyes with a fiber-optic light source; a puncture was created in the sclera at the tumor shadow for tissue excision. A tumor biopsy with the total volume of 12–18 mm^3^ was obtained directly from the enucleated globes of patients. Fresh surgical specimens were collected and processed as described previously [19]. Blood was drawn from patients for germline *RB1* mutation screening. All experimental protocols were approved by the Institutional Review Board at the Faculty of Medicine Ramathibodi Hospital, Mahidol University (protocol number ID11-58-53 and ID12-61-32) and performed in accordance with the relevant guidelines and regulations. Informed consent was obtained from a parent of each patient before the samples were collected.

### Retinoblastoma organoids

Four lines of organoids were previously established (RB668, RB654, RB170, and RB187) [18, 19]; the other line (RB394) was generated in this study from RB tissues. Derivation of RB organoids was performed as previously described [19]. In brief, RB tissues were dissociated, and tumor cells were resuspended in organoid medium and embedded in growth factor-reduced Matrigel solution (Corning) at a ratio of 1:1.8 by volume. Aliquots of 20 µL/well of the mixed cell-gel solution were added to 6-well plates via 5–7 drops/well and allowed to solidify at 37 °C. Organoid medium was added to cover the gel drops, and cultures were maintained in a humidified incubator 5% CO_2_ incubator at 37 °C. Every 3–4 weeks, organoids were manually dissociated and passaged at a 1:3 or 1:4 ratio by embedding in fresh Matrigel.

### Drugs

The Approved Oncology Drugs Set VIII, consisting of 133 FDA-approved chemotherapeutic drugs was kindly provided by the National Cancer Institute Division of Cancer Treatment and Diagnosis/ Developmental Therapeutics Program (http://dtp.cancer.gov) of the National Institutes of Health (Bethesda, MD, USA). Drugs were provided at concentrations of 10 mM in 100% dimethyl sulfoxide (DMSO). Stock plates were diluted in 96- or 384-well polypropylene V-bottom microplates using an automated JANUS liquid handling platform (Perkin Elmer).

### Primary drug screening and imaging-based drug response analysis

Tumor organoids were manually dissociated, incubated with TrypLE Express for 15 min at 37 °C, and resuspended in organoid medium with 5% Matrigel. Droplets of the cell/gel mixture (8 × 10^4^ cells/10 µL) were placed in the center of wells of a 96-well plate. After a thin gel had formed, medium was added and the cultures were maintained for 7 days. RB organoids were then exposed to drugs (10 µM) or vehicle (0.1% DMSO) for 72 h. To evaluate the drug response, viable and dead cells were distinguished by incubating the organoids with a mixture of the fluorescent DNA-binding dyes Hoechst 33342 (Invitrogen) and ethidium homodimer-1 (Abcam, 1:1000) for 15 min. Organoids were imaged before and after drug treatment using an Operetta plate imaging system (Perkin Elmer). The area of live organoid cells (Hoechst 33342-positive, ethidium homodimer-1−negative) was determined using a Columbus image data storage and analysis system. Normalized growth rate inhibition (GR) was calculated as previously described [25] (see Supplementary Methods for details). GR values range from − 1 to + 1, where negative values indicated cytotoxicity, a value of 0 reflected complete cytostasis, and positive values indicated partial growth inhibition.

### Secondary drug screening and dose-response curve analysis

Organoid cells were seeded in 384-well culture plates at 7.5 × 10^3^ cells/15 µL/well in organoid medium containing 5% Matrigel. A 6-point, 10-fold dilution series ranging from 0 to 100 µM or 200 µM was generated and added to the cells in duplicate using a JANUS liquid handler workstation. After 72 h, cell viability was quantified using a CellTiter-Glo (ATP) luminescent assay according to the manufacturer’s instructions (Promega). Dose-response curves were fitted using nonlinear least squares regression with variable slope (four parameters); half-maximal inhibitory concentration (IC_50_), the fraction of viable cells at the highest drug concentration (E_max_), and curve steepness (Hill Slope, HS) were computed using GraphPad Prism 8.

### Differentiation of hESCs into retinal organoids

Retinal organoids were generated according to a previously published protocol [26]. Briefly, the hESC cell line KhES-1 (RIKEN, Japan) was dissociated and rapidly aggregated in mTeSR1 medium (STEMCELL Technologies) containing 10 µM Y-27632 (Sigma-Aldrich) at 3 ×10^3^ cells/well in 96-well ultra-low adhesion V bottom Lipidure-coated plates (S-BIO). Differentiation media were added at specific time points as previously described, without the addition of γ-secretase inhibitor (DAPT) [26]. Retinal organoids were maintained for 65–70 days, examined for the expression of photoreceptor-associated genes, and used to evaluate retinal toxicity of the drugs.

### Drug treatments

Sunitinib, melphalan, topotecan, or vehicle (0.1% DMSO) was added at the indicated concentrations to organoid cultures and incubated for 24 h. Drug efficacy and toxicity were evaluated by immunostaining.

### Cell cycle analysis

Cell cycle was analyzed using 647 EdU click proliferation kit (BD Biosciences) in accordance with the manufacturer’s instructions. Briefly, organoid cells were incubated with 10 µM EdU for 90 min. Cells were fixed, permeabilized, and stained with EdU detection cocktail for 30 min. Cell suspensions were washed and incubated with propidium iodide for 15 min. Stained cells were immediately analyzed by flow cytometry (BD Biosciences). The data were analyzed using FlowJo software (BD Biosciences).

### Immunostaining

Retinal and tumor organoids were fixed with 3.7% and 2% paraformaldehyde solution, respectively for 10 min, washed with phosphate-buffered saline (PBS) and incubated in 30% (w/v) sucrose overnight, then embedded in OCT compound and snap frozen. Cryosections (10 µm) of samples were mounted on SuperFrost Plus slides for immunostaining. Cryosections were washed with PBS and stained with primary (overnight) and secondary antibodies. The following antibodies were used for staining: RXR gamma (1:100, mouse, sc-365252, Santa Cruz Biotechnology), Ki67 (1:100, rabbit, RB1510P0, Thermo Scientific™ Lab Vision™), c-Jun (1:400, rabbit, 9165, Cell Signaling Technology), and cleaved caspase 3 (1:400, rabbit, 9661, Cell Signaling Technology). Secondary antibodies (1:500) included Alexa Fluor 488 goat anti-mouse IgG and Alexa Fluor 568 goat anti-rabbit IgG (Invitrogen). Nuclei were counterstained by 4’,6-diamidino-2-phenylindole (DAPI). Fluorescent images were acquired by confocal laser scanning microscopy, and Z-stacking was performed with NIS-Element AR (Nikon).

### Western blotting

Tumor organoids were lysed using a lysis buffer containing 1x Halt protease and phosphatase inhibitor cocktail (Thermo Fisher Scientific). Briefly, 20 µg total protein was separated using 10% SDS-PAGE gels and transferred to PVDF membranes. The membranes were blocked with 5% non-fat dry milk solution and incubated overnight at 4°C with anti-c-Jun rabbit antibodies (1:1000, 9165, Cell Signaling Technology), followed by incubation with anti-rabbit secondary antibodies conjugated to HRP. The target proteins were detected using a chemiluminescent HRP detection reagent (Millipore). GAPDH on the same membranes was detected using anti-GAPDH HRP-conjugated antibodies. The signal intensity was analyzed using ImageLab software (Bio-Rad). The relative densitometry of c-Jun bands was expressed as a function of GAPDH band densitometry and reported.

### Statistical analysis

Differences in response to drugs (IC_50_) of RB organoids were evaluated using the extra-sum of squares F-test. Cell cycle and death analyses were evaluated with an unpaired t-test. Multiple groups were compared using one-way analysis of variance (ANOVA) followed by Tukey’s multiple comparisons test or Welch’s ANOVA (for unequal variance) followed by Tamhane’s T2 multiple comparisons test. Significance was considered at *P* < 0.05. All statistical data analysis was performed using Prism 8. Hierarchical clustering and visualization were performed using heatmap.2.

Full details of the genomic and RNA sequencing methods and analyses, reverse transcription-quantitative polymerase chain reaction (RT-qPCR), and cell death analysis are provided in Supplementary Methods.

## Results

### Tumor organoids maintain the genomic landscapes of the parental RB tissues

**Fig. 1:**
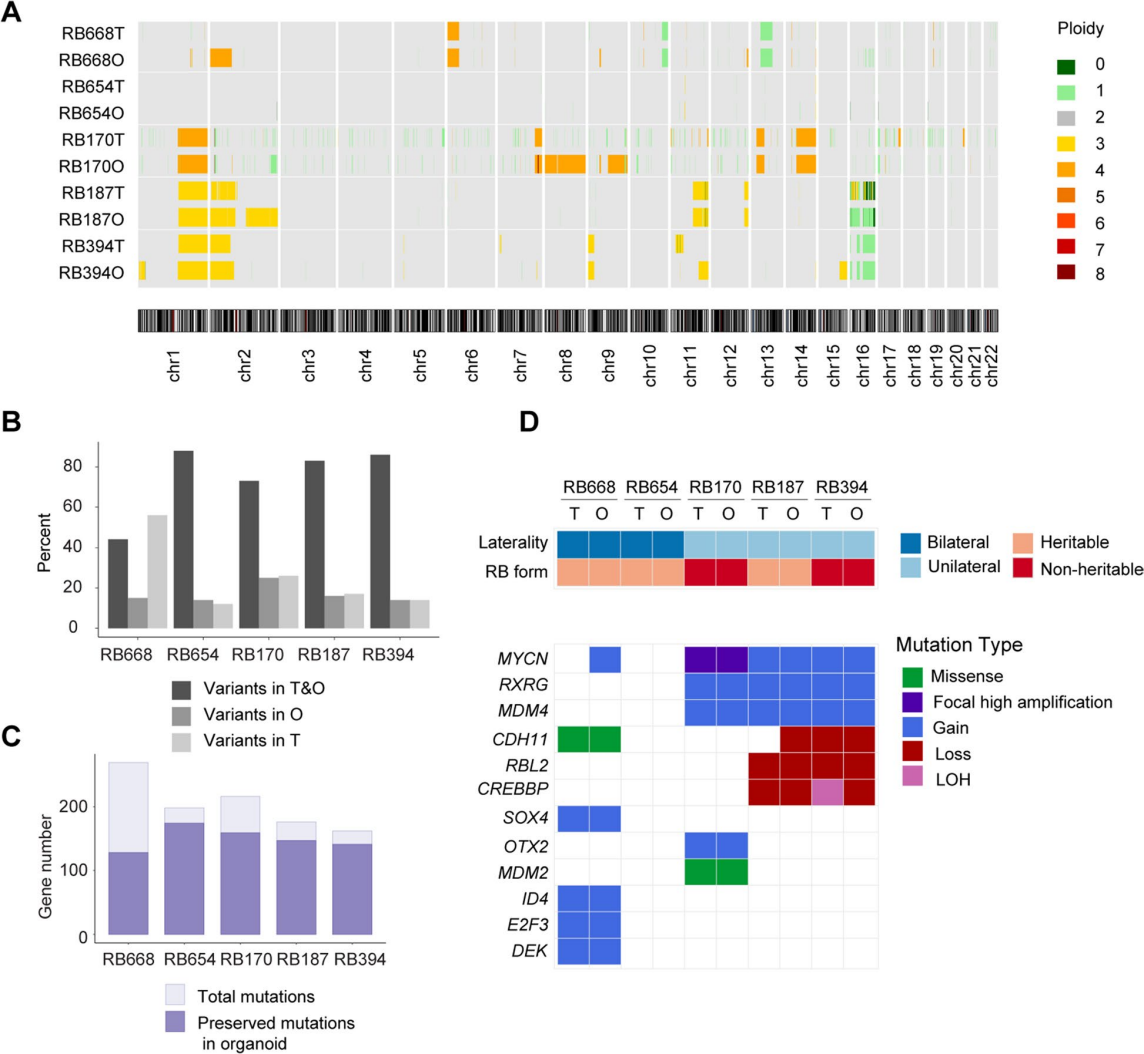
RB organoids retain the genomic landscape of the parental RB tissue. **A** Copy number variation profiles of retinoblastoma tissues (T) and the corresponding RB organoids (O) determined by comparative genomic hybridization/single nucleotide polymorphism array. **B, C** Pathogenic variants (**B**) and mutant genes (**C**) determined by whole-exome sequencing. **D** Summary of tumor laterality and RB forms, and commonly altered genes

Microarray data analysis of copy number variation showed that genomic alterations in the RB organoids were similar to those of the corresponding parental tissue from which they were derived (Fig. 1 A and Supplementary Fig. S1). The most common RB-associated alterations [4] identified in both tumor tissues and organoids were gains at chromosome 1q and/or 2p (RB668, RB170, RB187, and RB394), 16q loss (RB170, RB187, and RB394), and 6p gain (RB668). Unlike the other RB tissues/organoids, RB170, which was previously reported to have high-level focal *MYCN* amplification (90 copies) as the disease initiator [18], had chromosomal gain in 7q, 13q, and 14q. Gain or loss of smaller fragments was detected in each chromosome (Fig. 1 A and Supplementary Fig. S1). In contrast to the other four organoid/tissue pairs, RB654 exhibited minimal genomic alterations in tissue and organoid.

Of note, many subclonal mutations were further enriched in the organoids and were evident in chromosome 10q for RB688, chromosomes 2 and 16 for RB187, and chromosomes 1, 11q, 15, and 18 for RB394 (Supplementary Fig. S1). Furthermore, some lesions that were not detected in tumor tissues were identified in organoids (2p and 16q for RB668 and 8 and 9q for RB170), and conversely, some lesions previously detected in tumors were virtually absent in organoids, as shown for 7p and 11p of RB394 tissue and organoids (Fig. 1A and Supplementary Fig. S1). These findings imply that clonal evolution that results in tumor heterogeneity may occur in organoids, as previously described [15]. Analysis of whole-exome sequencing data indicated that more than 70% of pathogenic variants and mutant genes in the parental tumors were also present in the corresponding organoids for four of the pairs (Fig. 1B, C), but only 50% were maintained in RB668 organoids, possibly due to deletion of 13q and subsequent induction of significant genomic changes (Fig. 1A–C).

The five organoids were derived from patients with both heritable and non-heritable RB, as indicated by germline *RB1* mutation analysis (Fig. 1D). Four organoids (RB668, RB654, RB187 and RB394) represented *RB1*-deficient RB, and the other (RB170) was *MYCN*-amplified RB [18]. Many genes commonly altered in RB were identified, including those associated with 1q, 2p, and 6p gains and 16q loss (Fig. 1D) [4]. Additionally, mutations were identified in genes associated with cone signal circuitry that is oncogenically activated in RB [4]; namely, *MYCN* (RB668, RB170, RB187 and RB394), *RXRG* (RB170, RB187 and RB394) and *MDM2* (RB170). Taken together, these data demonstrate that RB organoids recapitulated the genomic landscapes of the parental tissues. We selected three organoid lines representing RB initiated by *MYCN* amplification (RB170) and biallelic *RB1* inactivation (RB654 with minimal lesions and RB668 with 13q deletion) (Fig. 1A–D) for the initial drug screening.

### High-throughput screening discriminates between cytotoxic and cytostatic effects of anticancer drugs on RB organoids

**Fig. 2:**
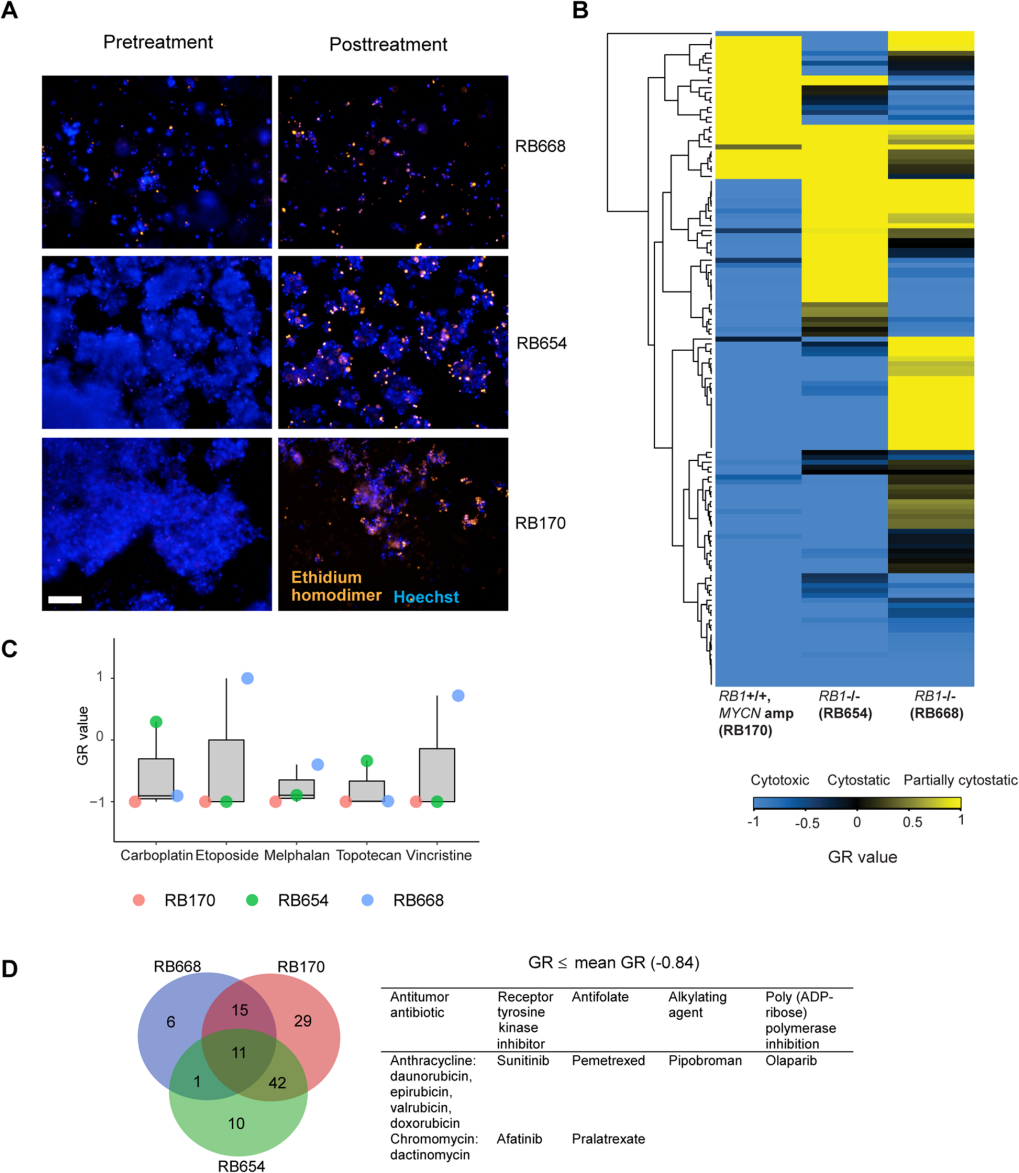
Primary screening identifies FDA-approved anticancer drugs with cytotoxicity towards RB organoids. **A** Representative fluorescence micrographs of organoids pre- and post-treatment with a cytotoxic drug. Hoechst (blue) stains all nuclei, whereas ethidium homodimer (orange) stains only dead cells. **B** Heat map of the growth rate inhibition (GR) values of single doses of drugs (10 µM) in three RB organoids. **C** GR values of clinically used anticancer drugs for RB. **D** Venn diagram of drugs with GR ≤ the mean GR value in three organoids. A total of 11 drugs inhibited the growth of all organoids. Scale bar: 100 μm (**A**)

Organoids were incubated for 72 h with vehicle (0.1% DMSO) or 133 FDA-approved compounds at 10 µM (Fig. 2A). Areas of live and dead organoid cells were quantified before and after drug exposure and used to calculate the normalized GR on a scale of − 1 to + 1, where negative and positive numbers represent cytotoxicity and partial growth inhibition, respectively, and 0 reflects complete cytostasis [25] (Fig. 2B). RB organoids exhibited heterogenous drug responses; ~78% of drugs were cytotoxic in RB170 organoids compared with 49% and 65% of drugs in RB668 and RB654 organoids, respectively (Fig. 2B). Among the drugs commonly used to treat RB, at least one organoid line was resistant to carboplatin, etoposide, and vincristine, whereas all three organoid lines were inhibited by melphalan and topotecan (Fig. 2C).

We then selected the drugs that were highly cytotoxic based on GR ≤ the mean GR (-0.84) in all organoid lines. We identified 11 drugs that were consistently highly cytotoxic in all three organoid lines; these included five antitumor antibiotics, two antifolates, two tyrosine kinase receptor inhibitors, an alkylating agent, and a poly (ADP-ribose) polymerase inhibitor (Fig. 2D). However, melphalan and topotecan appeared less cytotoxic in RB668 and RB654, respectively, as indicated by high GR compared with the mean GR and thus were excluded (Fig. 2C).

### Sunitinib is a potent inhibitor of RB organoid growth

**Fig. 3:**
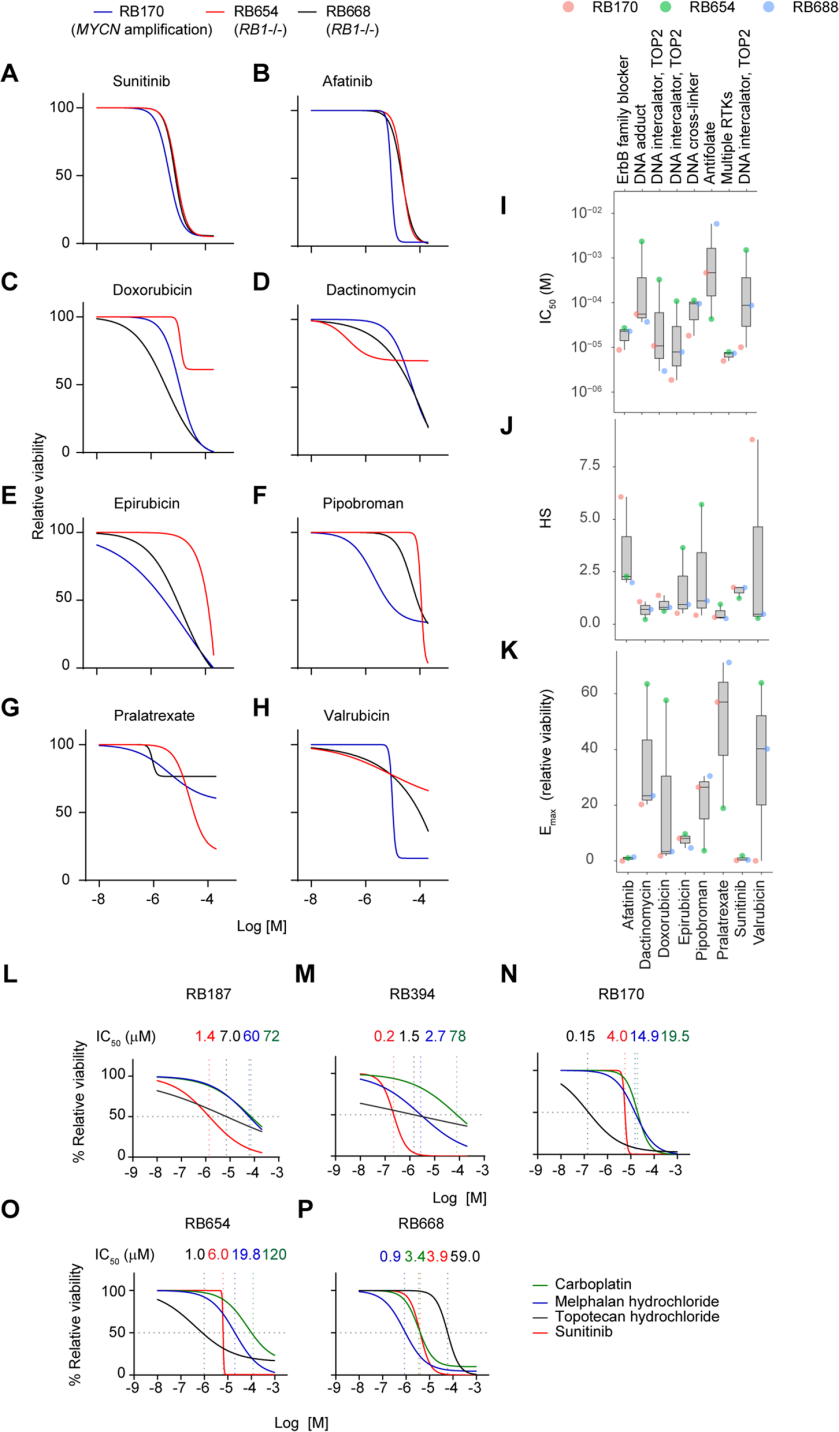
Secondary screening identifies sunitinib as a high-potency inhibitor of RB organoid growth. **A**–**H** Dose-response curves of 8 drugs tested on three organoids. **I**–**K** Data from A–H were used to calculate IC_50_ (**I**), HS (**J**), and E_max_ (**K**) values in the three RB organoids (colored circles). Boxes and whiskers indicate median values with interquartile ranges and variability outside the upper and lower quartiles, respectively. RTKs: receptor tyrosine kinases; TOP2: topoisomerase II. **L**–**P** Dose-response curves of sunitinib compared with the indicated drugs in five RB organoid lines

To evaluate the potency and efficacy of the 11 selected drugs, full dose-response matrices were generated for the three organoid lines (Supplementary Fig. S2A–C). Three drugs failed to achieve 50% cytotoxicity over the range of concentrations tested and were excluded based on low potency (Supplementary Fig. S2A–C). The dose-response curves of the remaining 8 drugs (Fig. 3A–H) were used to compute IC_50_, E_max_, and HS, which are measures of the potency, efficacy, and the degree of homogeneity of the drug response, respectively [27] (Fig. 3I–K). The matrices for the 8 drugs showed positive correlations between the IC_50_ and E_max_ and negative correlations between HS and both IC_50_ and E_max_, indicating that the drugs with high potency and efficacy induced a homogeneous response in the organoid cultures (Supplementary Fig. S2D).

We found that sunitinib, a multiple tyrosine kinase inhibitor, was the only drug that had an IC_50_ of < 10 µM in all three organoid lines [RB170 (*MYCN* amplification), RB654 (*RB1*-/-) and RB668 (*RB1*-/-)]; the drug responses were similar across the organoids (Fig. 3A, I). The responses to afatinib were also similar across the organoids, but the IC_50_ were > 10 µM in RB668 and RB654 (Fig. 3B, I). Additionally, sunitinib and afatinib had HS values > 1 in all three organoid lines, indicative of homogeneous responses (Fig. 3J). Sunitinib, afatinib, and epirubicin had low E_max_ values (< 10%) in the three organoid lines (Fig. 3K). Collectively, these data showed that sunitinib outperformed the other drugs tested in terms of potency (IC_50_), efficacy (E_max_), and response (HS) in all three organoids. We also compared sunitinib with the clinically used drugs carboplatin, melphalan, and topotecan in five organoid lines (RB187, RB394, RB170, RB654, and RB668). Sunitinib had consistently superior potency and efficacy compared with carboplatin and melphalan in four lines (RB187, RB394, RB170, and RB654) (Fig. 3L–P). When compared with topotecan sunitinib showed higher potency in three lines (RB187, RB394, and RB668) and higher efficacy in all lines (Fig. 3L–P). Additionally, sunitinib treatment provided for the most homogeneity of the drug response as indicated by curve steepness resulting in high HS values (Fig. 3L–P).

### Sunitinib signatures indicate the drug effects on proliferation and differentiation in RB

**Fig. 4:**
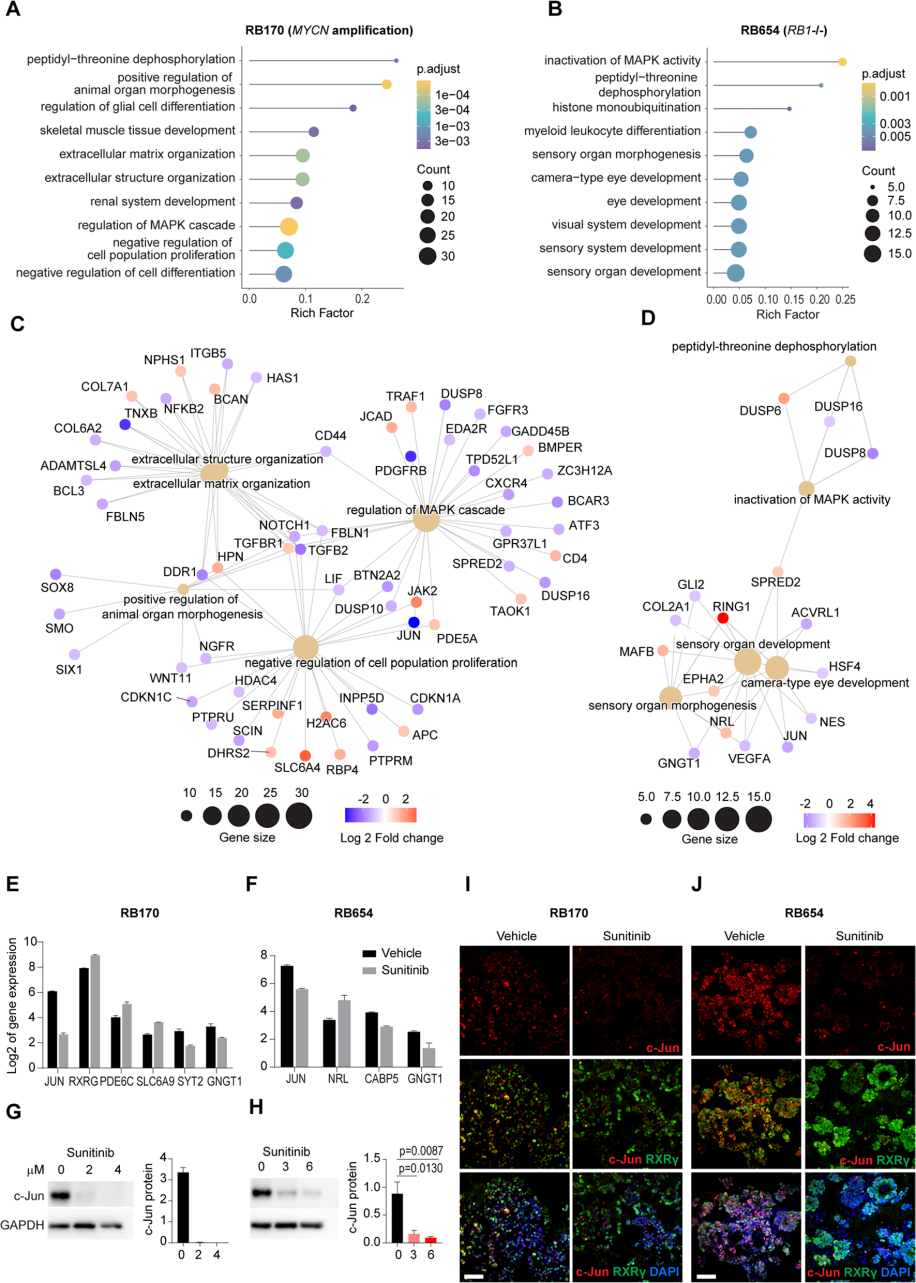
Gene Ontology (GO) analysis of the sunitinib gene signature in RB organoids. **A, B** The top 10 significant biological process terms enriched among differentially expressed genes (DEGs) of RB170 (**A**) and RB654 (**B**) organoids with sunitinib treatment at IC_10_ (2 µM and 3 µM, respectively) for 72 h. Rich factor is defined as the ratio of input DEGs with a given annotated term to all genes with that annotation. **C, D** Gene networks of the top five enriched GO terms after treatment of RB170 (**C**) and RB654 (**D**) organoids with sunitinib. **E, F** Expression of *JUN* and retina-associated genes from a set of DEGs from RB170 (**E**) and RB654 (**F**) organoids. **G, H** Protein expression of c-Jun detected by western blotting in RB170 (**G**) and RB654 (**H**) organoids treated with vehicle or sunitinib at IC_10_ and IC_50_ (4 µM and 6 µM, respectively) for 72 h. Data obtained from three independent experiments; means ± SEM are shown. **I, J** Representative staining of c-Jun (red), RXRG (green), and nuclei (DAPI, blue) in RB170 (**I**) and RB654 (**J**) organoids treated with vehicle or sunitinib at IC_50_. Image acquisition and processing was performed using the identical parameters. Scale bar: 50 μm (**I**, **J**)

To examine the action of sunitinib in RB tumors RB654 (*RB1*-/-) and RB170 (*MYCN* amplification) organoids reflecting on the case study of two RB subtypes initiated with different cancer driver genes were selected for downstream analyses. Sunitinib signatures were generated by evaluating the differentially expressed genes (DEGs) in sunitinib-treated vs. vehicle-treated RB organoids. The concentration used was about IC_10_, which retained 90% cell viability, to avoid excessive cell death (Supplementary Fig.S3A) and thus reflect the changes in gene expression of sunitinib-treated tumor cells. The sunitinib signatures of RB170 and RB654 comprised 422 and 194 DEGs, respectively. Gene Ontology (GO) analysis revealed that the most significantly enriched terms for both organoids were strongly associated with mitogen-activated protein kinase (MAPK) signaling (Fig. 4A, B). This finding was supported by additional Kyoto Encyclopedia of Genes and Genomes pathway analysis (Supplementary Fig. S3B, C).

Next, we analyzed gene-biological concept networks of the top five GO terms for RB170 and showed interconnection between “regulation of the MAPK cascade” and other terms related to negative regulation of proliferation and positive regulation of organ morphogenesis (Fig. 4 C). *JUN*, a component of the AP-1 transcription factor that regulates cell proliferation and survival [28], and *CD44*, a target of AP-1 [29], were dramatically downregulated by sunitinib treatment (Fig. 4C), suggesting that sunitinib exerted antiproliferative on RB organoids *via* regulation of MAPK signaling. Additionally, *NOTCH1* was connected to all five GO terms, and its downregulation is known to be associated with impairment of RB proliferation [30, 31].

The same analysis was performed for RB654 and showed that the term “inactivation of MAPK activity” was connected to three GO terms associated with “sensory organ/camera-type eye development” and “peptidyl-threonine dephosphorylation” (Fig. 4D). *JUN* was downregulated in RB654 by sunitinib treatment, whereas *NRL*, a transcription factor of rod photoreceptor fate [32] that is usually depleted in RB [4], was upregulated (Fig. 4D). Both *JUN* and *NRL* were included in the GO terms related to development, prompting us to speculate that sunitinib may promote tumor differentiation *via* inactivation of MAPK activity in RB654 organoids. Notably, *JUN* was the most significantly downregulated gene from tumor organoids of both RB subtypes (Fig. 4E, F). Downregulation of the *JUN* expression was confirmed by mRNA and protein analyses (Supplementary Fig. S3D and Fig. 4G, H). We found that c-Jun was co-expressed with RXRG cone marker; the levels of c-Jun were reduced in sunitinib-treated RB170 and RB654 cells (Fig. 4I, J). Examining retinal genes in a set of DEGs revealed that cone (*RXRG* and *PDE6C*) and amacrine (*SLC6A9* also expressed in cones) genes in RB170 and rod gene (*NRL*) in RB654 were upregulated after sunitinib treatment (Fig. 4E, F). In contrast, bipolar (*SYT2, CABP5*) and rod (*GNGT1*)-enriched genes were downregulated in organoid cells after sunitinib treatment (Fig. 4E, F). Taken together, sunitinib signatures suggest that the drug affected cell proliferation and differentiation in RB.

### Sunitinib suppresses RB proliferative tumor cones

**Fig. 5:**
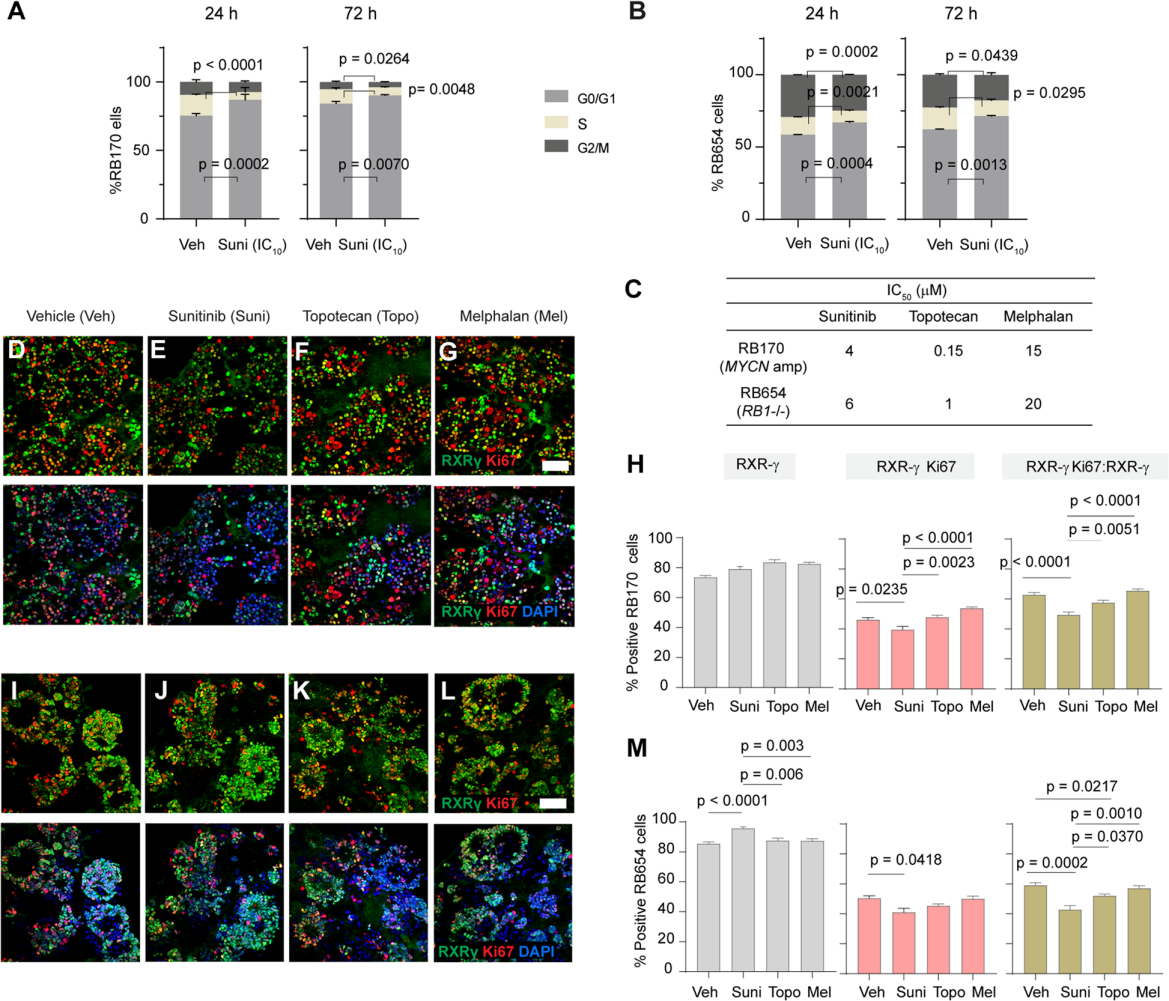
Sunitinib induces cell cycle arrest and suppresses proliferative tumor cones in RB organoids. **A, B** Percentage organoid cells in G0/G1, S, and G2/M cell cycle phases after sunitinib treatment at the IC_10_ (2 µM and 3 µM, respectively) of RB170 (**A**) and RB654 (**B**) for 24 or 72 h compared with vehicle. **C** IC_50_ of sunitinib, topotecan, and melphalan in RB170 and RB654 organoids. **D**–**G** Representative staining of RXRG (green), Ki67 (red), and nuclei (DAPI, blue) in RB170 organoids treated for 24 h with vehicle or the indicated drugs at their IC_50_. **H** Percentage non-cycling and cycling tumor cones and the proportion of proliferative tumor cones in RB170 calculated from the data shown in (**D**–**G**). **I**–**M** Same as (**D**–**H**) but for RB654 organoids. The number of RXRG + tumor cells (non-cycling) and RXRG + Ki67 + tumor cells (cycling) was determined from a total number of DAPI-positive tumor cells. The ratio of RXRG + Ki67 + cells to RXRG + cells indicated a proportion of proliferative tumor cones. Data (**A**, **B**, **H**, **M**) obtained from three independent experiments; means ± SEM are shown. For immunofluorescence data (**D**–**M**), 6 tumor organoid gel drops for each treatment in different experiments were used; about 6000 cells counted from 15 microscope fields (5 fields/experiment) for each treatment. Scale bar: 50 μm (**D–G**, **I–L**)

To support the findings from sunitinib signatures the effect of drug treatment on cell cycle progression was determined. The cell cycle analysis indicated that treatment of RB170 and RB654 organoids for 24 or 72 h with sunitinib induced significant G1 arrest and a reduction in cell numbers in S and M phases (Fig. 5 A, B), which is in line with the GO term related to antiproliferation (Fig. 4A, C).

Most tumor cells in RB have characteristics of cone photoreceptors, including the subpopulations that have ability to self-renew and initiate new RB in vivo [33–35]. To determine whether drugs suppressed proliferative tumor cones, thus inhibiting tumor growth, we examined co-localization of the cone-specific transcription factor RXRG [36], which is expressed in tumor cells of origin in both *RB1*^–/–^- and *MYCN*-driven RB subtypes [33, 37–39], and the proliferative marker Ki67 in RB170 and RB654 organoids treated with vehicle or selected drugs at their IC_50_ for 24 h (Fig. 5 C). We compared the effects of sunitinib on proliferative tumor cones with melphalan and topotecan.

Immunostaining of organoids revealed that a significant reduction in the proportion of proliferative tumor cones (RXRG^+^ Ki67^+^: RXRG^+^) was observed after treatment of RB170 organoids with sunitinib (49.29% ± 2.02%, mean ± SEM) compared with vehicle (62.71% ±1.57%), topotecan (57.43% ±1.67%) and melphalan (65.46% ± 1.23%; Fig. 5D–H). In contrast, topotecan and melphalan had no significant effects on proliferative tumor cones compared with vehicle (Fig. 5D, F–H). There was a slight increase in the number of non-cycling cells (RXRG^+^) after treatment of RB170 with the tested drugs, but only sunitinib significantly decreased the number of cycling cells (RXRG^+^ Ki67^+^), resulting in a reduced proportion of proliferative tumor cones (Fig. 5H).

The same analysis in RB654 organoids showed significant reductions in the proportion of proliferative tumor cones (RXRG^+^ Ki67^+^: RXRG^+^) following treatment with sunitinib (42.74% ± 2.72%) and topotecan (51.92% ± 1.28%) but no significant change with melphalan treatment (57.07% ± 1.71%) compared with vehicle (59.03% ± 1.79%) (Fig. 5I–M). Interestingly, the non-cycling cells (RXRG^+^) increased, whereas the cycling cells (RXRG^+^ Ki67^+^) decreased after only sunitinib treatment (Fig. 5M), making sunitinib more effective than either topotecan or melphalan as also observed with RB170. The findings from immunostaining analysis agreed with the results from cell cycle and drug signature analyses and indicated that sunitinib inhibited proliferation as shown by the number of cycling and non-cycling cells in RB organoids. Altogether, sunitinib was superior to the other tested drugs in terms of suppression of proliferative tumor cones.

### Sunitinib exhibits low toxicity in normal hESC-derived retinal organoids and has high efficacy against tumor organoids

**Fig. 6:**
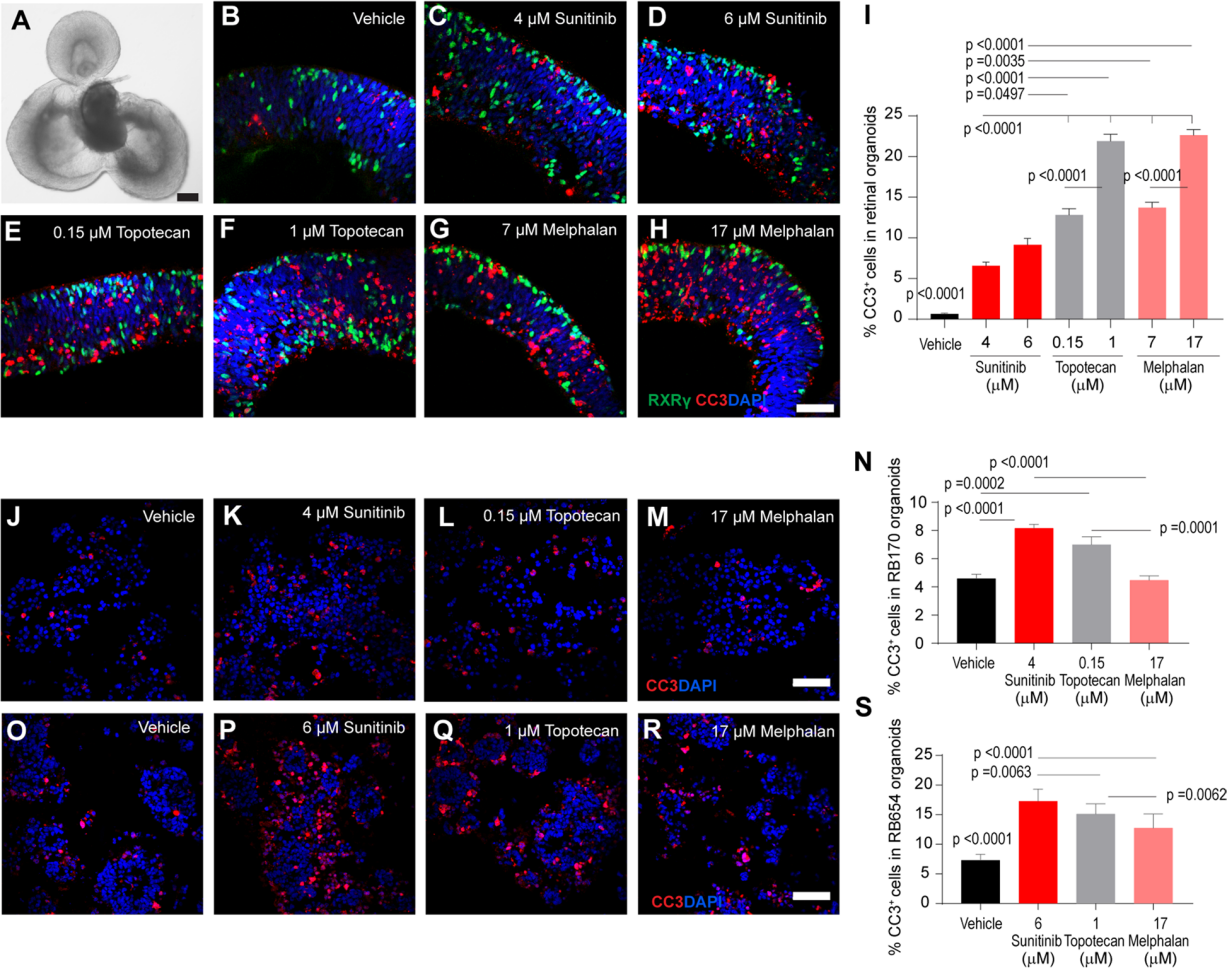
Sunitinib exerts minimal retinal toxicity and has high efficacy against RB compared with clinically used drugs. **A**–**H** Representative hESC-derived retinal organoids at day 70 of differentiation (**A**) and immunostaining of RXRG (green), cleaved caspase 3 (CC3, red), and nuclei (DAPI, blue) after treatment for 24 h with vehicle or the indicated drugs (**B**–**H**). **I** Percentage CC3-positive cells calculated from the data in (**B**–**H**). **J–M** Immunostaining of CC3 (red) and nuclei (DAPI, blue) after treatment for 24 h of RB170 with vehicle or the indicated drugs. **N** Percentage CC3-positive cells calculated from the data in (**J**–**M**). **O**–**S** Same as (**J**–**N**) but for RB654 organoids. The number of CC3-positive tumor cells was determined from a total number of DAPI-positive tumor cells. Data shown are obtained from three independent experiments; means ± SEM are shown. *p*-value of the vehicle in (**I**, **S**) refers to each comparison between vehicle and drug treatment. Six retinal organoids for each treatment in different experiments were used; about 6000 cells counted from 15 microscope fields (5 fields/experiment) for each treatment (**I**). Six tumor organoid gel drops for each drug treatment in different experiments were used; about 4500 cells counted from 12 microscope fields (4 fields/experiment) for each treatment (**N**, **S**). Scale bars: 200 μm (**A**), 50 μm (**B**–**H, J–M, O–R**)

To examine the potential retinal toxicity of sunitinib, we generated retinal organoids derived from hESCs, which represent a useful model of otherwise inaccessible human retina [40]. Retinal organoids staining positively with the RXRG cone marker (day 65–70; Fig. 6A–H) were incubated with vehicle, sunitinib, topotecan, or melphalan [at the IC_50_ for tumor organoids for 24 h (Fig. 5C)], and cell death was examined by immunostaining of cleaved caspase 3 (CC3), a marker of apoptosis. Under the identical conditions used for evaluation of drug efficacy, the percentage CC3^+^ cells in retina organoids were 0.66% ± 0.11% (mean ± SEM) for vehicle; 6.57% ± 0.44% and 9.15% ± 0.79% for 4 µM and 6 µM sunitinib, respectively; 12.82% ± 0.78% and 21.91% ± 0.83% for 0.15 µM and 1 µM topotecan, respectively; and 13.71% ± 0.68% and 22.63% ± 0.70% for 7 µM and 17 µM melphalan, respectively (Fig. 6B–I). Topotecan and melphalan were clearly highly toxic to the retinal organoids compared with both vehicle and sunitinib despite treatment with lower drug dose (< IC_50_ for 7 µM melphalan) (Fig. 6B–I).

Immunostaining of CC3 was conducted to examine the tumor killing effect of the same set of drugs under the identical conditions used for evaluating drug toxicity. The percentage CC3^+^ cells in RB170 organoids were 4.59 ± 0.31% (mean ± SEM) for vehicle; 8.16% ± 0.28% for sunitinib; 6.99% ± 0.56% for topotecan; and 4.47% ± 0.31% for melphalan (Fig. 6J–N). In RB654, the percentage CC3^+^ cells were 7.28 ± 0.29% for vehicle; 17.27% ± 0.59% for sunitinib; 15.11% ± 0.50% for topotecan; and 12.74% ± 0.68% for melphalan (Fig. 6O–S). Sunitinib was more effective than other drugs in killing RB tumor cells (Fig. 6N, S). Death cells from sunitinib treatment were more in tumor organoids but fewer in retinal organoids than those from topotecan and melphalan treatment (Fig. 6I, N, S). Thus, sunitinib displayed high efficacy against RB with minimal retinal toxicity compared with topotecan and melphalan.

## Discussion

A drug with improved antitumor activity and a favorable retinal safety profile is urgently needed to advance globe-sparing therapies in RB. In the present study, we sought to identify FDA-approved drugs that could potentially be repurposed for RB treatment. We performed high-throughput screening of a panel of 133 drugs and successfully identified sunitinib as a highly cytotoxic agent for RB. Sunitinib effectively suppressed proliferative tumor cones in RB organoids but had minimal toxicity towards normal retinal organoids, and outperformed melphalan and topotecan, which are used for local chemotherapy of RB.

RB organoids derived from both *RB1*-deficient and *MYCN*-amplified subtypes were used as a model for drug screening. *MYCN*-amplified organoids were generally more susceptible than *RB1*-deficient organoids to inhibition by the panel of anticancer drugs. High *MYCN* expression confers a rapid growth and proliferation phenotype [41, 42], which may explain this sensitivity to drugs that typically kill rapidly dividing cells. In line with this possibility, poorly differentiated cells in *MYCN*-amplified RB have been shown to be more responsive to chemotherapy than differentiated tumor cells in *RB1*-deficient RB with featured rosettes [4, 22]. Nevertheless, we showed that sunitinib exhibited anticancer activity against RB organoids of both subtypes in the present study.

Sunitinib has shown broad and potent antitumor and/or antiangiogenic activity in preclinical studies of multiple cancer types [43–45]. We demonstrated the antitumor activity of sunitinib in RB, consistent with the previous report on direct effect of sunitinib on inhibition of RB invasion and metastasis [46], although the effect on angiogenesis is inconclusive in orthotopic model of RB in zebrafish [46]. Sunitinib has been shown to induce G1-phase arrest in acute myeloid leukemia (AML) cells [45], similar to our observations here with RB, but this contrasts with the G2/M-phase arrest seen in sunitinib-treated glioblastoma cultures [44].

We speculate that sunitinib affects tumor proliferation and differentiation *via* downregulation of *JUN* for suppression of the RB growth. c-Jun is overexpressed in a large number of cancers [47], and its silencing has been shown to inhibit proliferation, migration, and invasion of cancer cells [48, 49]. Downregulation of *JUN* with a concomitant G1-phase arrest is consistent with the function of c-Jun in promoting G0 to G1 transition [49, 50] and indicates the antiproliferative effect of sunitinib in tumor organoids of both RB subtypes. Furthermore, high expression levels of c-Jun are linked to poorly differentiated cells in a high grade of malignancy in glial tumors [51], suggesting the role of c-Jun in inhibition of cancer differentiation. Downregulation of *JUN* with concomitant changes in expression of photoreceptor genes indicates sunitinib-induced RB differentiation in *MYCN*-amplified and *RB1*-deficient organoids. As indicated by the upregulation of cone-specific transcription factor *RXRG* [36], sunitinib induced cone differentiation in *MYCN*-amplified RB170 which has been classified as aggressive RB exhibiting poor differentiation and dedifferentiated cone states (subtype 2) [23]. *RB1*-deficient RB654, classified as differentiated tumors with high expression of cone genes (subtype 1) [23], underwent further differentiation by sunitinib treatment as shown by the upregulation of a critical transcription factor *NRL* which is essential for rod differentiation [32]. An additional supporting evidence for sunitinib-induced RB differentiation is an increase in non-cycling cells and decrease in cycling cells that result in a reduced proportion of proliferative tumor cones in RB organoids. RB that is turned into more differentiated states might be less aggressive by sunitinib treatment, as previously described in human AML blast cells undergoing sunitinib-elicited monocytic differentiation [45], providing a potential strategy for the cancer treatment.

Intravitreal or intra-arterial chemotherapy has increased the rate of globe retention in globe-preservation treatment and significantly reduces drug-induced systemic toxicity [5, 10]. However, drug-induced retinal toxicity remains a major drawback of local chemotherapy. Melphalan and topotecan have been reported to cause retinal toxicity, as measured by reductions in electroretinography [8–12], particularly when administered intravitreally alone or in combination with intra-arterial chemotherapy for the management of vitreous disease in RB. This means that the choice of chemotherapeutic agents is crucial to advance the treatment. Our study suggests that sunitinib has potential utility for local chemotherapy for RB given its high efficacy in terms of suppressing proliferative tumor cones and minimal retinal toxicity compared with other drugs currently in use.

Patient-derived tumor organoids and hESC-derived retinal organoids as a 3-dimensional model are of use to drug discovery. Additionally, previous work has shown that RB-like retinal organoids can be generated from *RB1*-/- hESC and iPSC cells and allow for the possibility of testing therapeutic agents [38, 39]. However, in vivo models may be needed to verify antineoplastic effects and retinal safety of drugs. Our study provides the groundwork for and encourages further investigation of the therapeutic potential of sunitinib for local chemotherapy of RB. In a preclinical model, sunitinib has been shown to have a good retinal safety profile when administered by intravitreal injection [52], which supports the potential utility of locally delivered sunitinib for treating RB.

## Conclusion

In the present study, we utilized RB organoids derived from different RB subtypes to screen for drugs that could be repurposed for RB. We identified sunitinib as a possible drug for RB. Sunitinib exhibits high efficacy with minimal retinal toxicity, suggesting that it may be suitable for local therapy. Sunitinib is FDA-approved for the treatment of renal carcinoma and imatinib-refractory gastrointestinal stromal tumors, and our study identifies RB as a potential new indication.

## Supplementary Information

**Additional file 1:** **Supplementary Methods. ** for genomic and RNA sequencing analyses, cell death analysis, and RT-qPCR. **Supplementary Figure S1.** Copy number alterations and loss of heterozygosity by using the comparative genomic hybridization/single nucleotide polymorphism array. B-allelic frequency (BAF) and log R-ratio (LRR) plots indicate genomic alterations in tumor tissue (T) and the corresponding organoid (O). **Supplementary Figure S2.** Secondary drug screening assay: A–C Dose-response curves of 11 drugs tested in RB organoids. D Correlation coefficients of dose-response matrixes of 8 drugs tested in three organoid lines were computed by Pearson’s correlation at a significant level of 0.05. *P = 0.0337 for IC_50_ and E_max_; * *P* = 0.0352 for E_max_ and HS; *** *P* = 4.885 × 10^− 15^ for E_max_ and area under the curve (AUC). **Supplementary Figure S3.** Cell death and pathway analyses: A Cell death analysis by annexin V-FITC and PI staining of RB170 and RB654 organoid cells. B, C KEGG pathway analysis of gene signature of sunitinib treatment in RB170 (B) and RB654 (C) organoids. D *JUN* mRNA expression in organoids treated with vehicle or sunitinib at IC_10_ and IC_50_ for 72 h.

## Data Availability

All data generated or analyzed during this study are included in this published. article (and its supplementary information files). The datasets generated during and/or analyzed during the current study are available in the Gene Expression Omnibus (GEO) repository, [https://www.ncbi.nlm.nih.gov/geo/query/acc.cgi?acc=GSE201740]

## Abbreviations

IC_50_: Half-maximal inhibitory concentration
E_max_: The fraction of viable cells at the highest drug concentration
HS: Hill Slope
GR: Growth rate inhibition
RB: Retinoblastoma
hESCs: Human embryonic stem cells
DEGs: Differentially expressed genes
*RB1*: RB transcriptional corepressor 1
*MYCN*: MYCN proto-oncogene, BHLH transcription factor
*RXRG*: Retinoid X receptor gamma
*NR**L*: Neural retina leucine zipper
*MDM2*: MDM2 proto-oncogene
*JUN*: Jun proto-oncogene, AP-1 transcription factor subunit
Ki67: Marker of proliferation Ki-67
