# Supplementary material for: Sunitinib efficacy with minimal toxicity in patient-derived retinoblastoma organoids
Source: J Exp Clin Cancer Res. 2023 Feb 1;42:39. doi: 10.1186/s13046-023-02608-1 (PMC9890748; doi:10.1186/s13046-023-02608-1)
Supplement: Supplementary file 1 — Additional file 1: Supplementary Methods. for genomic and RNA sequencing analyses, cell death analysis, and RT-qPCR. Supplementary Figure S1. Copy number alterations and loss of heterozygosity by using the comparative genomic hybridization/single nucleotide polymorphism array. B-allelic frequency (BAF) and log R-ratio (LRR) plots indicate genomic alterations in tumor tissue (T) and the corresponding organoid (O). Supplementary Figure S2. Secondary drug screening assay: A–C Dose-response curves of 11 drugs tested in RB organoids. D Correlation coefficients of dose-response matrixes of 8 drugs tested in three organoid lines were computed by Pearson’s correlation at a significant level of 0.05. *P = 0.0337 for IC50 and Emax; * P = 0.0352 for Emax and HS; *** P = 4.885 × 10− 15 for Emax and area under the curve (AUC). Supplementary Figure S3. Cell death and pathway analyses: A Cell death analysis by annexin V-FITC and PI staining of RB170 and RB654 organoid cells. B, C KEGG pathway analysis of gene signature of sunitinib treatment in RB170 (B) and RB654 (C) organoids. D JUN mRNA expression in organoids treated with vehicle or sunitinib at IC10 and IC50 for 72 h. [file 13046_2023_2608_MOESM1_ESM.docx]

# Sunitinib efficacy with minimal toxicity in patient-derived retinoblastoma organoids

**Supplementary Methods**

**Calculation of the normalized growth rate inhibition (GR)**

GR was computed according to the following equation reported as previously described [1].

$$GR=2^{\frac{{log}_{2}\left( \frac{X\left( c \right)}{X_{0}} \right)}{{log}_{2}(\frac{X_{ctrl}}{X_{0}})}}-1$$

The X(c) and X_ctrl_ were the mean areas of live organoid cells (Hoechst 33342-positive, ethidium homodimer-1−negative) in drug-treated organoids and in DMSO-treated organoids, respectively at the end of treatment procedure. The X_0_ was the mean area of live organoid cells measured just prior to drug treatment. GR values range from −1 to +1, where negative values indicate cytotoxicity, 0 reflects complete cytostasis, and positive values indicate partial growth inhibition.

**Genomic analysis**

DNA from tumor tissue, RB organoids, and blood was extracted as previously described [2]. Copy number alterations and loss of heterozygosity were determined by using the comparative genomic hybridization/single nucleotide polymorphism array (Infinium CytoSNP-850Kv1-2_iScan_D1, Illumina) in accordance with the manufacturer’s instructions. Normalized signal intensity and genotype were computed using Illumina’s GenomeStudio v.2 software. Individual CNV was detected using cnvPartition CNV Analysis Plug-In v3.2.0 in GenomeStudio data analysis software. For whole-exome sequencing, exons were captured using SureSelect Human All Exon v7 (Agilent Technologies) for library preparation. The libraries were sequenced using an Illumina platform; the read length and throughput depth were 150 bp and 160×, respectively. Somatic variants and small indels were called using GATK4 Mutect2 using the tumor-only mode where the panel of normal was generated from whole-exome sequencing data of 47 heathy Thais (unpublished data). The single nucleotide variants and small indels were annotated using Variant Effect Predictor (VEP; version 105; https://asia.ensembl.org/info/docs/tools/vep/). Variants with population allele frequency <1% in any populations from 1000 Genome Phase 3 (https://www.internationalgenome.org/category/phase-3/) were selected. The impact of variants was predicted using metaSVM [3], metaLR, FATHMM [4], SIFT [5], and PolyPhen2 [6].

Mutation was examined in *RB1* of blood DNA from the RB patients by using Sanger sequencing and multiplex ligation-dependent probe amplification as previously described [7].

**RNA-sequencing**

RNA extraction and the measurement of RNA quality and quantity were conducted as previously described [8]. Specimens with an RNA integrity number (RIN) >8.0 were used in this study. RNA Libraries were constructed by using the TruSeq Stranded mRNA LT Sample Prep Kit (Illumina Inc.), in accordance with the manufacturer’s instructions. RNA sequencing was performed with the Illumina NovaSeq sequencing system (100-bp paired-end reads); an average of 41 million read counts were generated for all samples.

Processing RNA-seq Reads:

Low-quality reads were purged using Trimmomatic (v0.36) [9]. Quantification of the transcriptome was carried out using Kallisto (v0.43) [10] using annotation from Ensembl version 84 at the transcript level. Gene level counts were generated using the tximport R package [11], and gene count level data were normalized using the TMM (trimmed mean of M-values) method followed by CPM (counts per million) computation. RNA-seq data were deposited in Gene Expression Omnibus (GEO) through accession number GSE201740.

Differential Expression Analysis:

Differential expression analysis between sample groups was performed using exactTest function as implemented in the edgeR R package [12]. Genes were selected based on a log2 fold-change value ≥1 and adjusted p-value ≤.01.

Gene Ontology Analysis:

Gene ontology (GO) for differentially expressed genes was performed using enrichGO function and visualized using dot plot and gene-concept network in clusterProfiler 4.0 R package [13].

**Cell death analysis**

Cell death was analyzed using annexin V–fluorescein isothiocyanate (FITC) apoptosis detection kit (BioLegend, San Diego, CA) in accordance with the manufacturer’s instructions. Briefly, dissociated cells were washed and incubated with annexin V-FITC and propidium iodide (PI) for 15 min. Stained cells were immediately analyzed by flow cytometry. The data were analyzed using FlowJo software.

**Reverse transcription-quantitative polymerase chain reaction (RT-qPCR)**

Total RNA was extracted from cell cultures using TRIZOL reagent (Invitrogen). DNA was removed from RNA by RQ1 RNase-Free DNase, and 1 µg RNA was then reverse-transcribed into cDNA using an Improm-IITM reverse transcriptase system supplemented with 100 µg/mL random primer (Promega), 3 mM MgCl_2_, and 0.5 mM dNTP. The thermal cycling condition of reverse transcription (RT) reaction consisted of annealing (25°C for 5 min), extension (42°C for 1 h), and reverse transcriptase inactivation (70°C for 15 min). RT-qPCR was performed using SYBR green master mix (Bio-Rad) on CFX96 Touch Real-Time PCR Detection System (Bio-Rad) with the thermal cycling condition as follows: initial denaturation (95°C for 20s) and 40 cycles of two-step thermal profile (95°C for 5s and 60°C for 30s), followed by melting curve analysis. Target gene expression levels was normalized to β -actin expression levels. Primer sequences were listed as follows: c-Jun_F: AAAGGAAGCTGGAGAGAATCG, c-Jun_R: TGTTTAAGCTGTGCCACCTG, Human β-actin_F: GGCACCCAGCACAATGAAGATC, and Human β-actin_R: GTAACGCAACTAAGTCATAGTCCGC.


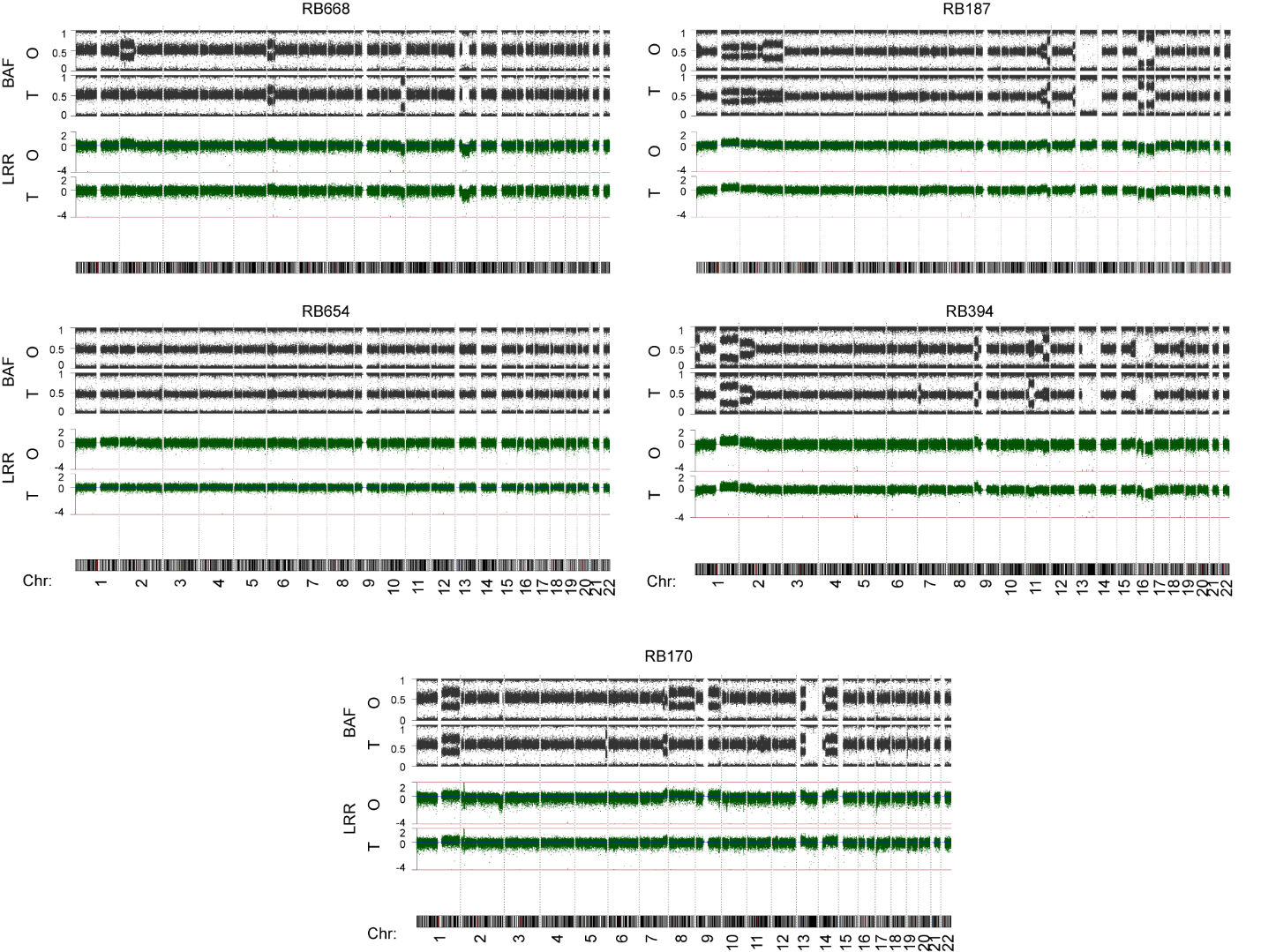


**Supplementary Fig. S1** Copy number alterations and loss of heterozygosity by using the comparative genomic hybridization/single nucleotide polymorphism array. B-allelic frequency (BAF) and log R-ratio (LRR) plots indicate genomic alterations in tumor tissue (T) and the corresponding organoid (O)


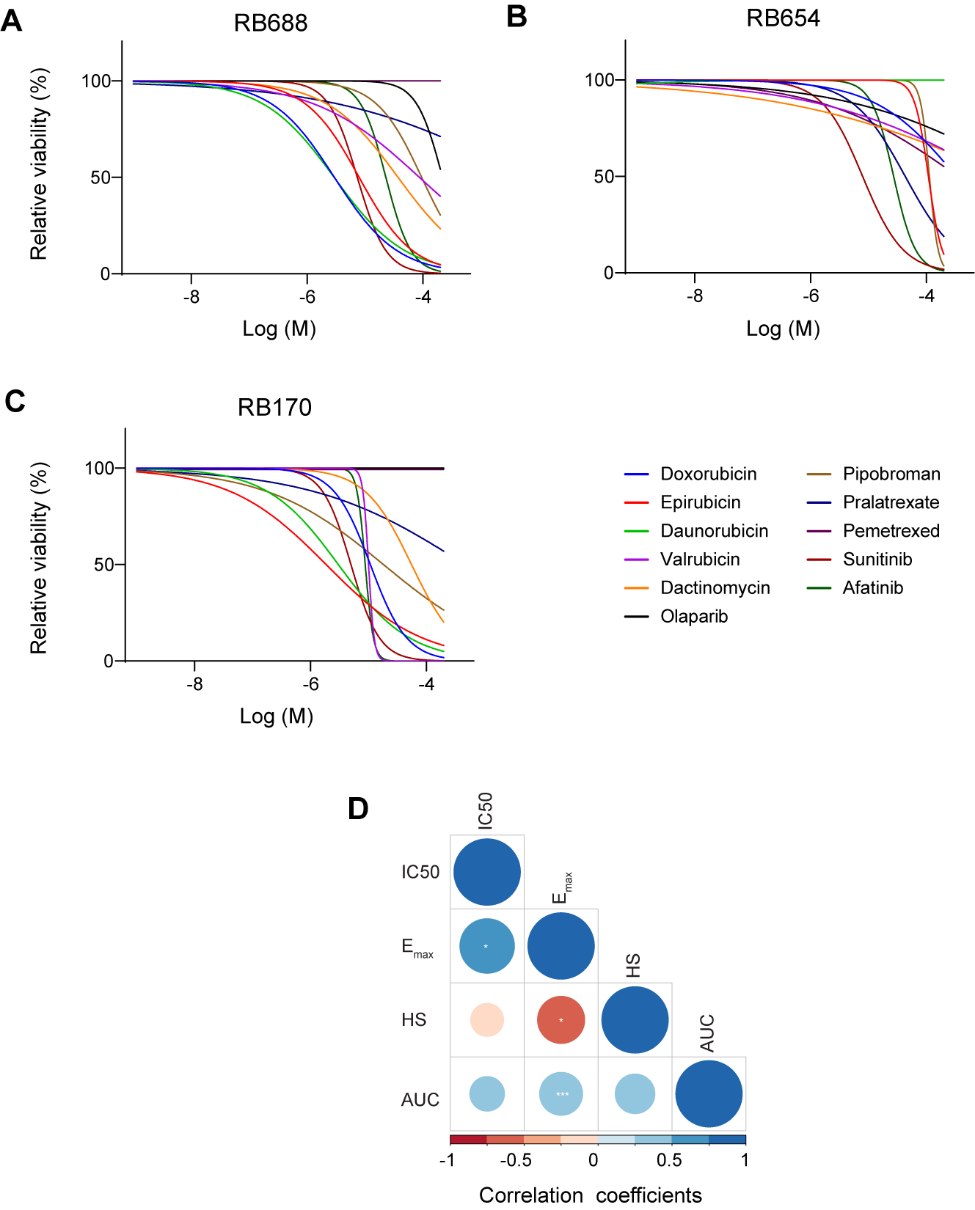


**Supplementary Fig. S2** Secondary drug screening assay

**A**–**C** Dose-response curves of 11 drugs tested in RB organoids. **D** Correlation coefficients of dose-response matrixes of 8 drugs tested in three organoid lines were computed by Pearson's correlation at a significant level of 0.05. **P* = 0.0337 for IC_50_ and E_max_; **P* = 0.0352 for E_max_ and HS; *** *P* = 4.885 x 10^-15^ for E_max_ and area under the curve (AUC).


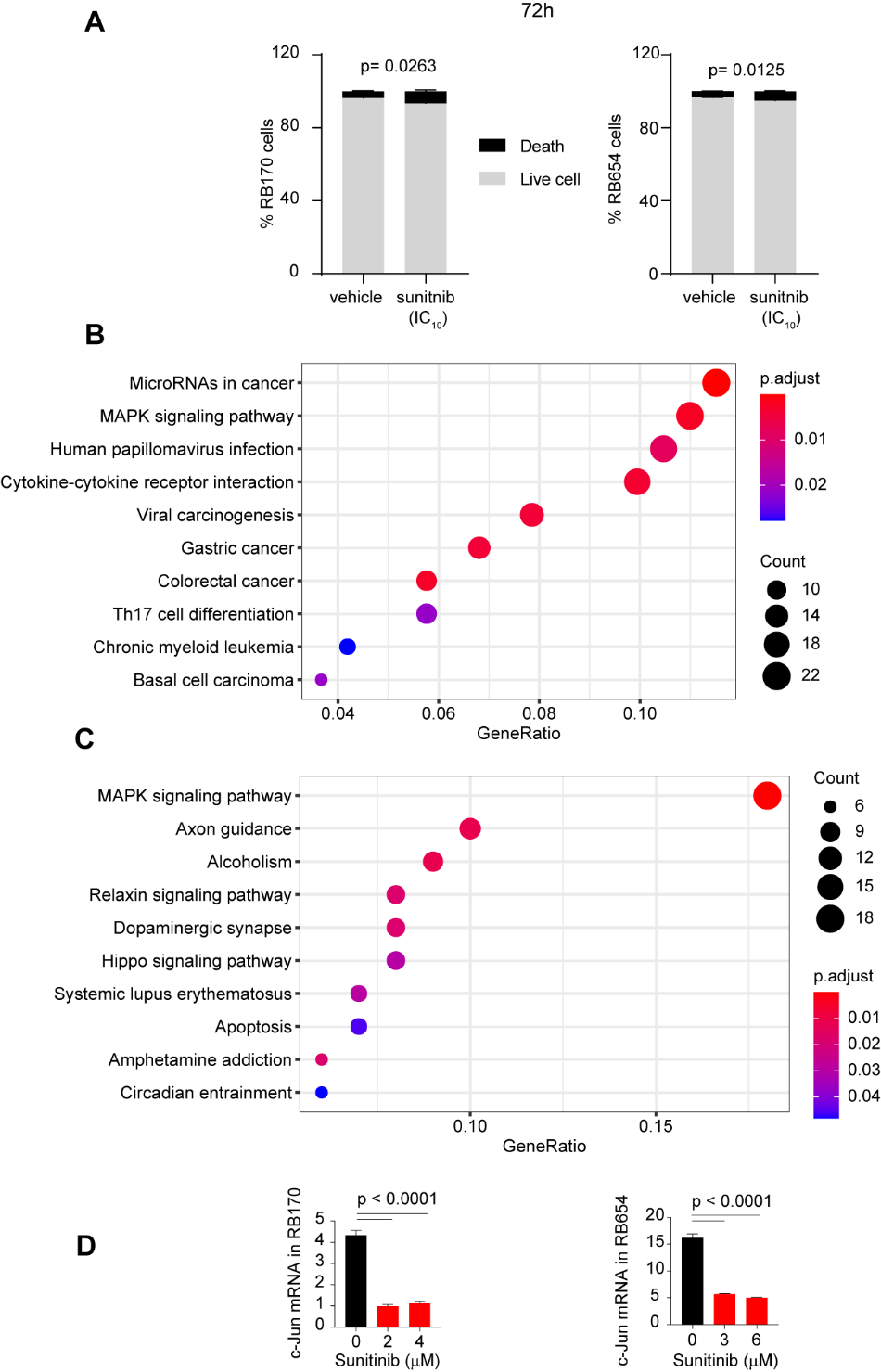


**Supplementary Fig. S3** Cell death and pathway analyses

**A** Cell death analysis by annexin V-FITC and PI staining of RB170 and RB654 organoid cells. **B**, **C** KEGG pathway analysis of gene signature of sunitinib treatment in RB170 (**B**) and RB654 (**C**) organoids. **D** *JUN* mRNA expression in organoids treated with vehicle or sunitinib at IC_10_ and IC_50_ for 72h.

**Supplementary References**

1. Hafner M, Niepel M, Chung M, Sorger PK. Growth rate inhibition metrics correct for confounders in measuring sensitivity to cancer drugs. Nat Methods. 2016;13(6):521–7.

2. Saengwimol D, Rojanaporn D, Chaitankar V, Chittavanich P, Aroonroch R, Boontawon T, et al. A three-dimensional organoid model recapitulates tumorigenic aspects and drug responses of advanced human retinoblastoma. Sci Rep. 2018;8(1):15664.

3. Kim S, Jhong JH, Lee J, Koo JY. Meta-analytic support vector machine for integrating multiple omics data. BioData Min*.* 2017;10:2.

4. Shihab HA, Gough J, Cooper DN, Stenson PD, Barker GL, Edwards KJ, et al. Predicting the functional, molecular, and phenotypic consequences of amino acid substitutions using hidden Markov Models. Hum Mutat. 2013;34(1):57–65.

5. Ng PC, Henikoff S. SIFT: predicting amino acid changes that affect protein function. Nucleic Acids Res. 2003;31(13):3812–14.

6. Adzhubei I, Jordan DM, Sunyaev SR. Predicting functional effect of human missense mutations using PolyPhen-2. Curr Protoc Hum Genet*.* 2013;Chapter 7:Unit7.20.

7. Rojanaporn D, Boontawon T, Chareonsirisuthigul T, Thanapanpanich O, Attaseth T, Saengwimol D, et al. Spectrum of germline RB1 mutations and clinical manifestations in retinoblastoma patients from Thailand. Mol Vis. 2018;24:778–88.

8. Saengwimol D, Chittavanich P, Laosillapacharoen N, Srimongkol A, Chaitankar V, Rojanaporn D, et al. Silencing of the long noncoding RNA MYCNOS1 suppresses activity of MYCN-amplified retinoblastoma without RB1 mutation. Invest Ophthalmol Vis Sci. 2020;61(14):8.

9. Bolger AM, Lohse M, Usadel B. Trimmomatic: a flexible trimmer for Illumina sequence data. Bioinformatics. 2014;30(15):2114–120.

10. Bray NL, Pimentel H, Melsted P, Pachter L. Near-optimal probabilistic RNA-seq quantification. Nat Biotechnol*.* 2016;34(5):525–27.

11. Soneson C, Love MI, Robinson MD. Differential analyses for RNA-seq: transcript-level estimates improve gene-level inferences. F1000Res. 2015;4:1521.

12. Robinson MD, McCarthy DJ, Smyth GK. edgeR: a Bioconductor package for differential expression analysis of digital gene expression data. Bioinformatics. 2010;26(1):139–40.

13. Wu T, Hu E, Xu S, et al. clusterProfiler 4.0: A universal enrichment tool for interpreting omics data. Innovation (N Y). 2021;2(3):100141.
